# A Comprehensive Characterization of the Differences in Meat Quality, Nonvolatile and Volatile Flavor Substances Between Taoyuan Black and Duroc Pigs

**DOI:** 10.3390/foods14111935

**Published:** 2025-05-29

**Authors:** Hanjing Shi, Sisi Chen, Wenyue Zhou, Junfei Xu, Zekun Yang, Liu Guo, Qilong Li, Qiuping Guo, Yehui Duan, Jianzhong Li, Fengna Li

**Affiliations:** 1 Hunan Provincial Key Laboratory of Animal Nutritional Physiology and Metabolic Process, Key Laboratory of Agro-Ecological Processes in Subtropical Region, Institute of Subtropical Agriculture, Chinese Academy of Sciences, Changsha 410125, China; shihj1015@163.com (H.S.); 17267896682@163.com (S.C.); guoiu20@mails.ucas.ac.cn (L.G.); liqilong23@mails.ucas.ac.cn (Q.L.); guoqiuping@isa.ac.cn (Q.G.); duanyehui@isa.ac.cn (Y.D.); 2 Hunan Provincial Key Laboratory of Animal Intestinal Function and Regulation, College of Life Sciences, Hunan Normal University, Changsha 410081, China; 3 College of Advanced Agricultural Sciences, University of Chinese Academy of Sciences, Beijing 100049, China; 4 College of Animal Science and Technology, Hunan Agricultural University, Changsha 410128, China; 15873173739@163.com (W.Z.); 15367941257@163.com (J.X.); 5 College of Animal Science and Technology, Guangxi University, Nanning 530004, China; 17300223772@163.com

**Keywords:** pork, meat quality, flavor quality

## Abstract

To compare the differences in meat quality between obese-type Chinese pig breeds and lean-type foreign pig breeds, we selected Taoyuan Black (TB) pigs and Duroc pigs at 180 and 210 days of age and analyzed their meat quality, chemical composition, and flavor compounds using an electronic tongue, chromatographic techniques, and two-dimensional gas chromatography-time-of-flight-mass-spectrometry (GC×GC-TOF-MS). A total of 16 main fatty acids, 18 main free amino acids, and 249 flavor compounds were identified. The results showed that TB pigs exhibited redder meat color, higher intramuscular fat, and lower shear force than Duroc pigs (*p* < 0.05). TB pigs displayed higher levels of flavor nucleotides, free amino acids, and monounsaturated fatty acids (*p* < 0.05). Furthermore, pigs at 180 days exhibited lower dripping loss and more flavor compounds than those at 210 days (*p* < 0.05). Electronic tongue analysis revealed higher umami values in TB pigs at 180 days of age. Among the flavor compounds in pork, the four compounds that contributed most significantly to flavor across all species were 2-nonenal, 2-octenal, heptanal, 2,3-butanedione, and 2-pentylfuran. These findings provide fundamental data and insight into pig production.

## 1. Introduction

China is the largest producer and consumer of pork in the world. Recent socioeconomic development has driven a paradigm shift in consumer preferences, with increasing emphasis on pork quality rather than mere availability. The quality and flavor of pork has increasingly become focal points of interest for consumers, determining the market value of pork [1]. The volatile and nonvolatile flavor substances of meat are influenced by various factors, including breed, age, sex, type of feed, and rearing mode, with breed and age being the primary causes of these differences [2]. Slaughter age can affect pork quality, the tenderness of pork decreases as the slaughter age of crossbred pigs increases, while the proportion of n-3 polyunsaturated fatty acids in finishing pigs may potentially rise with increasing slaughter age [3]. Researchers have extensively studied Shaziling pigs of different ages to explore the profiles of muscle amino acids, fatty acids, and metabolites to meat quality and found that age significantly affects dripping loss, shear force, and intramuscular fat (IMF) content of the *longissimus dorsi* (LD) muscle in Shaziling pigs [4].

Due to the unique genetic backgrounds of pig breeds, determining the optimal slaughter time requires consideration of the differences among various pig breeds. These differences not only influence nutritional quality, including the composition of fatty acids, but also affect the sensory quality of pork, making them important standards for evaluating meat quality [5,6]. For example, one study revealed significant differences in fatty acid composition between two pig breeds, with Nanyang pigs exhibiting higher MUFAs and a lower PUFA n-6/n-3 ratio than Landrace pigs [7]. Another study demonstrated that consumers prefer cooked loins from local pig breeds to those from hybrids during sensory evaluation [8]. Flavor is widely regarded as a key factor in consumer appeal and is characterized by qualitative and quantitative information on flavor compounds. Therefore, studying the diversity of flavor compounds in pork and their formation pathways is crucial, as these factors contribute to flavor discrepancies between different pig breeds. Researchers studied three different pig breeds and identified 79 volatile compounds, 15 of which were selected as odorants in pork [9]. Researchers have compared the nutritional and Chinese pork flavor profiles of five different breeds and found that 3-hydroxy-2-butanone (with a creamy aroma) is a characteristic flavor compound in Tibetan black pork [10].

The Duroc pigs are a widely breed international lean-type pig, known for its high litter size and fast growth rate. Available evidence shows that compared to Large White and Landrace pigs, the meat of Duroc pig has a better flavor but poorer taste [11]. Additionally, the meat of Chinese native pigs scores higher in sensory evaluations than those of Landrace × Duroc crossbred pigs [11]. The TB pig, a typical indigenous Chinese breed known for obese-type pigs, has been proven to possess unique advantages in muscle metabolic characteristics and muscle fiber type composition [12]. In recent years, Duroc and TB pigs have been studied by analyzing developmental changes in piglet immunity, and their findings suggest differences in the development of immune function among various pig breeds [13]. However, there are currently almost no studies comparing the meat quality, sensory evaluations, and volatile compounds of these two breeds.

In this study, we selected Duroc and TB pigs as research subjects to comprehensively compare meat quality and non-volatile and volatile flavor substances between these two pig breeds. Additionally, GC×GC-TOF-MS technology was used to identify the key volatile odor compounds in pork, providing fundamental data and insights for pig production.

## 2. Materials and Methods

### 2.1. Material

Twenty Duroc pigs (Yunyang County Zailing Agricultural Development Co., Ltd., Chongqing, China) and twenty TB pigs (Huisheng Taoyuan Black Pig Breeding Co., Ltd., Taoyuan County, China) at 30 days of age were selected as experimental materials, and each pig was kept in a single pen and fed accordingly. All pigs in the experiment were boars and were fed a uniform basal diet designed according to the NRC (2012) [14] from 30 to 210 days throughout the experiment. Ten pigs of each breed were randomly selected and slaughtered at 180 and 210 days. Pigs were stunned and exsanguinated according to standard industrial protocols. The animal experiments were approved by the Professional Animal Health Committee of the Institute of Subtropical Agricultural Ecology, Chinese Academy of Sciences (No. ISA-2022-0001). Animal experiments complied with the requirements of animal ethics and welfare. After slaughter, a sample of the LD muscle (including three vertebrae) was removed from the area between the 6th and 7th ribs on the back of the pig; some were placed in ice packs, while others were stored in liquid nitrogen, and subsequently all were kept in freezers at 4 °C or −80 °C for further analysis, respectively.

### 2.2. Meat Quality Analysis

Color measurements, including Lightness (L*), redness (a*) and yellowness (b*), were performed on each LD muscle sample at 24 h postmortem using a Chroma Meter CR-410 (Konica Minolta Sensing Inc., Osaka, Japan). Three determinations were carried out and the mean values were reported. Additionally, pH values were measured at three locations along the same muscle sample at both 45 min and 24 h using a pH meter (MATTHAUS, Potsdam, Germany). For drip loss determination, about 20 g of each sample was placed in a drip container (KABE Labortechnik, Elsenroth, Germany) singly, weighed and then stored at 4 °C. About 20 g of the sample was placed into a cooking bag and cooked in a water bath at 78 °C, using a temperature probe to monitor the internal temperature of meat until it reached 72 °C. These samples were weighed to calculate cooking loss rate.

For shear force determination, 100 g portions were similarly cooked as above. After reaching the target temperature, samples were cooled to room temperature and cut into 1.0 × 1.0 × 2.0 cm^3^ chunks with the longest dimension parallel to muscle fibers. These chunks were placed into a FTC TMS PRO texture analyzer (Food Technology Corporation, Hertfordshire, UK), and the blade was moved down at 1.5 mm/s, applying a force of 40 g. The device program generates stress-time curves and determines the shear force, and denoted by N.

For water-holding capacity determination, 100 g portions were similarly cooked and cooled as above. A circular meat pillar with a diameter of 2.5 cm and 1 cm was taken along the vertical direction of the muscle fiber, and 35 kg of pressure was applied using a M10 meat pressure meter ( Bulader, Beijing, China). The pressure loss was measured for 5 min, and then the hydraulic power was calculated by the formula:

Pressure loss rate (%) = [(Initial weight − Pressurized weight)/Initial weight] × 100

Water holding capacity (%) = [(Moisture − Pressure loss rate)/Moisture] × 100

### 2.3. Electronic Tongue Analysis

According to the suggestion of Zhao et al. [15], the electronic tongue procedure for pork analysis was adapted. An amount of 20 g of the cooked sample was accurately weighed and extracted by mixing with 100 mL of water using a blender. The mixed solution was centrifuged at 3000 rpm for 10 min, and the supernatant was analyzed using an electronic tongue INSENT SA402B Plus EX instrument (Intelligent Sensor Technology Inc., Kanagawa, Japan). Each sample underwent three replicate measurements, of which the final mean were used for analysis.

### 2.4. Chemical Composition Analysis

Approximately 50 g of fresh LD muscle was weighed, freeze-dried for 72 h, and reweighed. The moisture content of each sample was calculated as the difference between the initial and final weights. The freeze-dried samples were then ground using a hammer mill, and the samples (approximately 0.07 g of samples were analyzed using a Elementar Various MAX Cube carbon-nitrogen-sulfur elemental analyzer (Elementar, Langenselbold, Germany). The nitrogen content of each sample was multiplied by a factor of 6.25 to calculate the percentage of crude protein. The IMF content was analyzed according to the AOAC (2005) method.

### 2.5. Sapidity Nucleotides Analysis

Fresh LD muscle (0.5 g) was weighed in a centrifuge tube, and beads and 4 mL of perchloric acid solution (6 mol/L) were added to grind the sample at maximum speed for 3 min. After centrifugation at 6000 rpm for 10 min, the top layer of the liquid was moved to a cuvette, and a perchloric acid solution (6 mol/L) was added until the mixture reached 5 mL. Then, 2.5 mL of this solution was placed in another cuvette, and the pH of the mixture was adjusted to 7 using a 1 mol/L potassium hydroxide solution. The solution was diluted to 10 mL with a neutral aqueous solution. The top layer liquid was passed through a 0.22 μm filter membrane for analysis, and the solution was tested using a Agilent LC1290 high-performance liquid chromatography (Agilent, Santa Clara, CA, USA). The chromatographic column used was Zorbax NH2 (4.6 × 250 mm, 5 μm), with an injection volume of 10 μL and a flow rate of 1 mL/min. The column temperature was maintained at 25 °C, the mobile phase was 100% phosphate buffer, and the detection wavelength was set at 210 nm.

### 2.6. Flavor Amino Acid Analysis

After grinding the fresh sample (0.5 g), it was placed in a centrifuge tube, and 5 mL of hydrochloric acid (0.01 mol/L) was added to the tube. After centrifuging at 5000 rpm for 5 min, 0.5 mL of the liquid supernatant was taken and blended with 0.5 mL of 8% sulfosalicylic acid, then vortexed and kept at 4 °C overnight. After centrifuging at 12,000 rpm for 10 min, the supernatant was aspirated, and centrifugation was repeated until no precipitate remained. The solution was analyzed using a Hitachi L8900 amino acid analyzer (Hitachi High-Tech Inc., Hitachinaka, Japan). Chromatographic separation was conducted using an AccQ-Tag Ultra C18 column (Waters Corporation, Milford, MA, USA) (1.7 μm, 2.1 mm × 100 mm). The mobile phases used were as follows: phase A, AccQ-tag eluent A; phase B, 10% AccQ-tag eluent B; phase C, water; and phase D, AccQ-tag eluent B. The column temperature was maintained at 43 °C with a flow rate of 0.7 mL/min.

### 2.7. Fatty Acid Analysis

Approximately 0.5 g of freeze-dried LD muscle was accurately weighed into a centrifuge tube. Subsequently, a benzene-petroleum ether mixture (1:1 *v*/*v*) was added to the centrifuge tube and sealed for 24 h. Subsequently, the original volume was replenished with a benzene and petroleum mixture. Next, 5 mL of potassium hydroxide in methanol solution (0.4 mol/L) was added to the centrifuge tube, vortexed for 3 min, and allowed to sit for 30 min. Subsequently, 3 mL of ultrapure water was added, followed by vortexing for 3 min. The solution was then centrifuged at 3000 rpm for 3 min. Approximately 2 g of anhydrous sodium sulfate was added and allowed to stand for 5 min to remove excess water. Next, 0.2 mL of the upper layer of the solution was transferred to a 2 mL centrifuge tube and diluted with 0.8 mL of n-hexane. The solution was analyzed via liquid injection using a Agilent 7890A gas chromatograph (Agilent, Santa Clara, CA, USA).

A capillary column with a polydimethylsiloxane stationary phase (0.2 μm, 0.25 mm × 100 mm internal diameter) was used for chromatographic separation. The detector temperature was set to 280 °C, and the injector temperature was maintained at 270 °C. The column temperature was maintained between 100 °C and 180 °C.

### 2.8. Head-Space Solid-Phase Microextraction (HS-SPME)

An internal standard (ISTD) solution (10 mg/L) was prepared using a mixture of n-Hexyl-d13 and 50% ethanol and stored in a refrigerator at 4 °C. A saturated alkane solution (1 mg/L) was prepared by diluting n-Hexane and stored in a refrigerator at 4 °C. Three grams of the fresh sample were taken and placed into a centrifuge tube. The centrifuge tube was then frozen in liquid nitrogen for 5 min. Next, 1 g of the crushed sample was taken and placed into a 20 mL headspace vial, and 10 μL of ISTD solution was added to the vial. The sample was incubated at 80 °C for 10 min. Before extracting the sample, the SPME fiber was preconditioned by heating it in a chamber at 270 °C for 10 min. The SPME fiber was subsequently placed in the incubator at 80 °C for 40 min to facilitate extraction. After extraction, the SPME fiber was desorbed in the GC injector at 250 °C for 5 min. Following desorption, the SPME fiber was reconditioned by returning it to the chamber at 270 °C for 10 min. Additionally, a new 20 mL headspace vial was prepared by transferring 10 μL of Saturated Alkanes into it for further incubation, extraction, and injection processes. This sequence ensured proper handling and analysis of the sample via SPME and GC.

### 2.9. GC × GC-TOF-MS

The GC × GC analysis was performed using a LECO Pegasus BT 4D system (LECO, St. Joseph, MI, USA), equipped with a split/splitless injector and a dual-stage cryogenic modulator, coupled to a time-of-flight mass spectrometry (TOFMS) detector. The first-dimension column (1D): DB-Heavy Wax (30 m × 250 μm I.D., 0.5 μm) (Agilent, Santa Clara, CA, USA); second-dimension column (2D): Rxi-5Sil MS (2.0 m × 150 μm I.D., 0.15 μm) (Restek, Bellefonte, PA, USA). High-purity helium (>99.999%) served as the carrier gas, with a constant flow rate of 1.0 mL/min. Oven temperature program: initially held at 50 °C for 2 min, then increased to 220 °C at a rate of 4 °C/min, and maintained at this temperature for 13 min. The GC injector temperature: 250 °C. The mass spectrometry settings were as follows: flavor substances were analyzed using the LECO Pegasus BT 4D (LECO Inc., St. Joseph, MI, USA). The transfer line and TOF MS ion source were both kept at 250 °C. Spectra were acquired at a frequency of 200 spectra per second. The mass spectrometer was operated in EI mode at 70 eV, with a mass range of *m*/*z* 35–550 and a detector voltage of 2036 V.

### 2.10. Determination of ROAV

The relative odds activity value (ROAV) method was applied to assess the flavor profile of the sample [16]. ROAVmax refers to the flavor compound in the sample that is the most beneficial and is set as 100. The ROAVs for other flavor compounds were determined using the following formula:

ROAV = (C_A_/T_A_) × (T_stan_/C_stan_) × 100

In this context, C_A_ indicates the relative percentage content of a specific compound, while T_A_ is the threshold concentration of that compound in mg/kg. C_stan_ represents the relative percentage content of the compound with the highest flavor contribution, and T_stan_ denotes the threshold concentration (mg/kg) of that key compound. A compound is deemed a key flavor and odor-active substance when ROAV ≥ 1; the higher the ROAV, the greater its contribution to the overall flavor of the sample.

### 2.11. Identification and Quantification of Volatile Compounds

The identification of volatile compounds in pork was achieved by comparing their linear retention indices (RI) and retention times with the NIST2020 database [17]. Following this, assay data underwent comprehensive analysis using Chroma TOF and Classyfire [18], along with additional resources such as PubChem [19], Odor [20], and FlavorDB [21]. This project used an internal standard for normalization.2.12. Statistical Analysis

Flavor compound data were analyzed using ChromaTOF v5.x software to obtain information including the name of each sample substance, retention time, database retention index (RI) information, CAS number, actual retention index calculated for normal alkanes (C7–C30), and the peak area of the sample (*n* = 6). In this project, internal standard normalization was employed to standardize the data. A two-factor variance analysis (2 × 2 factorial analysis) was used to analyze the data of meat quality, chemical composition, sapidity nucleotides analysis, fatty acids, and amino acids with SPSS software (version 29.0), which includes age, variety, and their interaction. The data are presented as mean and SEM (n = 10); significant differences are indicated by *p* < 0.05, while a trend towards a difference is suggested when 0.05 < *p* ≤ 0.10.

## 3. Results

### 3.1. Analysis of Meat Quality and Chemical Composition

As shown in Table 1, from 180 to 210 days of age, the L* and pH_45min_ values of the LD muscle significantly decreased in TB and Duroc pigs, while the a*, b*, cooking loss, dripping loss values and moisture content significantly increased (*p* < 0.05). Comparing the pork samples with different breeds at the same age, it was found that the pH45min value, water-holding capacity and IMF content of TB pigs were significantly higher than those in Duroc pigs, whereas the b*, shear force, cooking loss, and dripping loss values were significantly lower in TB pigs compared to Duroc pigs (*p* < 0.05).

There is an interaction between age and breed on the L* value of the LD muscle (*p* < 0.05).

### 3.2. Analysis of Sensory Evaluation and Electronic Tongue

**Figure 1:**
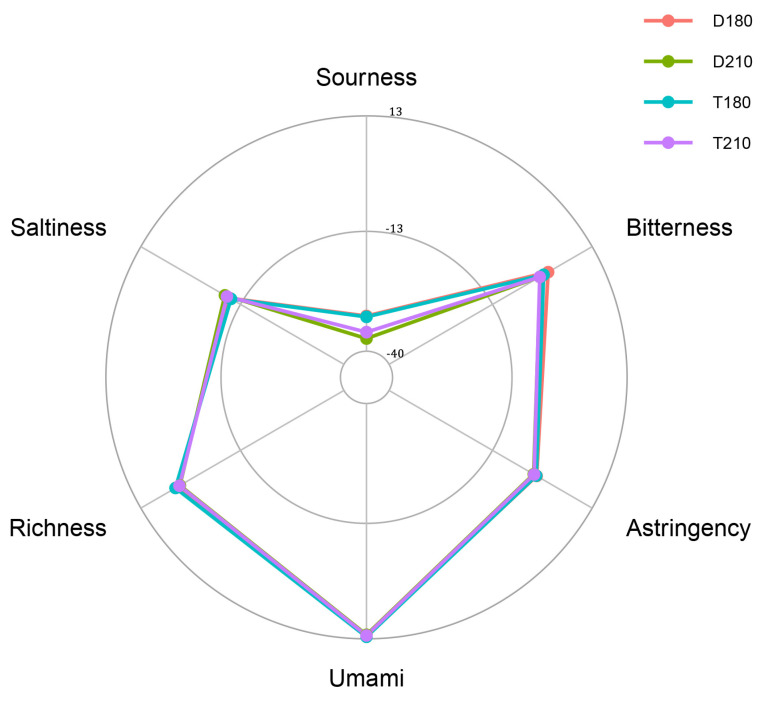
Electronic tongue determination of pork score. Different pork samples of electronic tongue sensors values radar map. D180, T180, D210 and T210 represent 180-day-old Duroc, 180-day-old TB pig, 210-day-old Duroc and 210-day-old TB pig.

As shown in Figure 1 and Table 2, the umami and richness scores of the four pork groups are higher than the baseline (bland point), with the highest values observed in the 180-day-old TB pigs. However, the Bitterness scores of the 180-day-old TB pigs and 180-day-old Duroc pigs are higher than the baseline, with the highest value observed in the 180-day-old Duroc pigs (*p* < 0.05).

### 3.3. Analysis of Flavor Amino Acid and Sapidity Nucleotides

As shown in Table 3, from 180 to 210 days of age, the contents of Glu, Ser, Thr, Ala, Tyr, Ile, Leu, Phe, and Trp, as well as total amino acids and most other amino acids in the LD muscle of TB and Duroc pigs significantly decreased (*p* < 0.05). Comparing pork samples of different breeds of the same age, the levels of Glu, Ser, Thr, Ala, Tyr, Ile, Leu, Phe, Trp, and total amino acids were significantly higher in TB pigs than in Duroc pigs (*p* < 0.05).

As shown in Table 4, the GMP and AMP contents in the LD of TB pigs were significantly higher than those in the Duroc pigs (*p* < 0.05).

There was an interaction effect between age and breed on the content of 15 free amino acids, excluding Ser, Asp, and Ala, in LD muscle (*p* < 0.05).

### 3.4. Analysis of Fatty Acid Profile

As shown in Table 5. From 180 to 210 days of age, the contents of saturated fatty acids C10:0, C12:0, C17:0, ∑SFA, ∑MUFA and the n-6: n-3 ratio decreased in the LD muscles both in TB and Duroc pigs, while the levels of polyunsaturated fatty acids including C18:3n3, and n-3 PUFAs increased (*p* < 0.05). Between the two breeds, the contents of ∑SFA and ∑MUFA in the TB pigs were significantly higher than those in Duroc pigs, while the ∑PUFA and n-6: n-3 ratio were significantly lower in the TB pigs compared to Duroc pigs (*p* < 0.05). There was an interaction effect between age and breed on the contents of C10:0 and C18:1n9t in the muscle of the pigs (*p* < 0.05).

### 3.5. Analysis of Volatile Flavor Compounds

#### 3.5.1. Volatile Flavor Substance Chromatography Analysis

**Figure 2:**
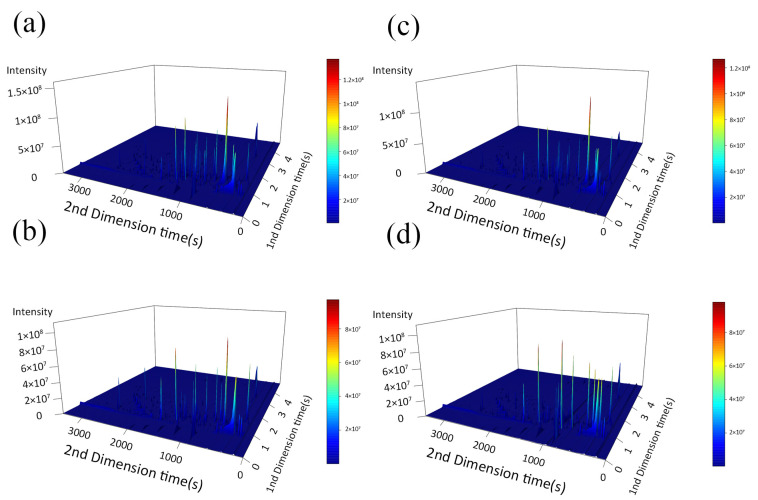
**Three-dimensional** Total Ion Chromatogram. (**a**) D180; (**b**) D210; (**c**) T180; (**d**) T210. D180, T180, D210 and T210 represent 180-day-old Duroc, 180-day-old TB pig, 210-day-old Duroc and 210-day-old TB pig, respectively.

The raw data of flavor compounds were systematically processed using the ChromaTOF software, and sensory annotation was performed in combination with the NIST2020 database to ensure the accuracy and reliability of compound identification. As shown in Figure 2, each sample showed excellent peak emergence, indicating a high concentration of volatile compounds in the LD muscle. Compared to the Duroc pig, the 3D Total Ion Chromatogram of the peak performance of the TB pig was higher and more complex. Through noise filtering and baseline correction of the raw data, we successfully obtained detailed identification results for flavor compounds.

#### 3.5.2. Quantity and Type of Volatile Flavor Substances Analysis

**Figure 3:**
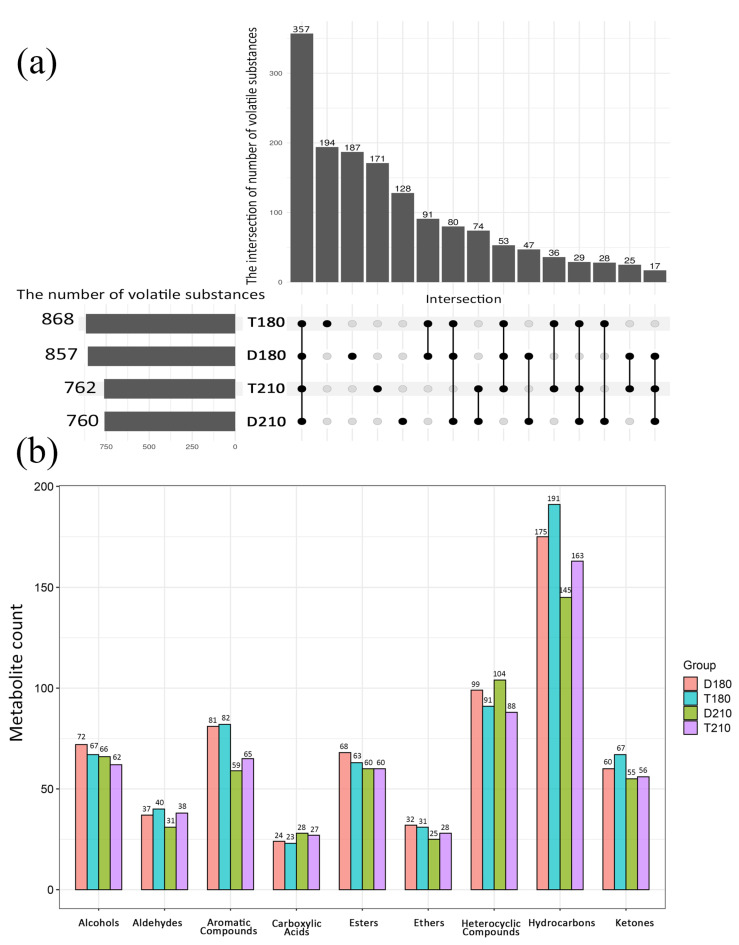
Metabolite Count and Venn Diagram. (**a**) Metabolite Count and amount of flavor compound overlaps of the different groups. The overlapping part represents the number of flavor substances identified jointly by multiple sample groups, and the non-overlapping part represents the number of flavor substances identified uniquely in the corresponding sample group. (**b**) Type of volatile flavor compounds in LD muscle of each group.

After filtering out noise, the identification details of the flavor compounds were obtained. In total, 1518 volatile flavor compounds were identified. As seen in Figure 3a, the 180-day-old TB pigs had the highest number of identified flavor compounds, reaching 868 species, followed by 180-day-old Duroc pigs (857 species), 210-day-old TB pigs (762 species), and 210-day-old Duroc pigs (760 species). Further analysis revealed that 357 flavor compounds were common to the four groups of samples, and these common compounds may be the main contributors to the base flavor of pork. As shown in Figure 3b, volatile flavor compounds of the LD muscle in each group were mainly identified as hydrocarbons, heterocyclic compounds, aromatic compounds, alcohols, esters, ketones, aldehydes, ethers, and carboxylic acids. Hydrocarbons were the most abundant category of compounds in all four pork samples, whereas Carboxylic Acids were the least abundant. The 180-day-old TB pigs contained the highest number of Hydrocarbons, Aldehydes, and Aromatic Compounds, whereas 180-day-old Duroc pigs contained the highest levels of Alcohols and Esters.

#### 3.5.3. ROAV Analysis

We systematically evaluated the contribution of key flavor compounds and their odor signatures in pork samples of different breeds and days of age by ROAVs analysis (Supplementary Data S1). The 180-day-old Duroc pigs were used as the reference, with 2-pentylfuran as the reference compound. In 180-day-old TB pigs, 210-day-old Duroc pigs and 210-day-old TB pigs, 2,3-butanedione was also used as the reference compound. As shown in Table 6, 2-Ethylhexanol, presenting a green and rose-like odor, was predominantly expressed in 180-day-old pork, with the highest ROAV in meat of TB pigs. Pentanal and heptanal, described as having repulsive and rancid odors, had the highest ROAVs in meat of 180-day-old Duroc, and it decreased with age. 2-Nonenal and 2-octenal mainly exhibited fatty odors, with the highest ROAVs in meat of 180-day-old Duroc pigs, followed by 180-day-old TB pigs, 210-day-old Duroc pigs and 210-day-old TB pigs. 1-Octen-3-one, primarily presenting a mushroom flavor, was predominantly expressed in 180-day-old meat, and with the highest ROAV in the meat of 180-day-old Duroc pigs.

#### 3.5.4. Multivariate Statistical Analysis

**Figure 4:**
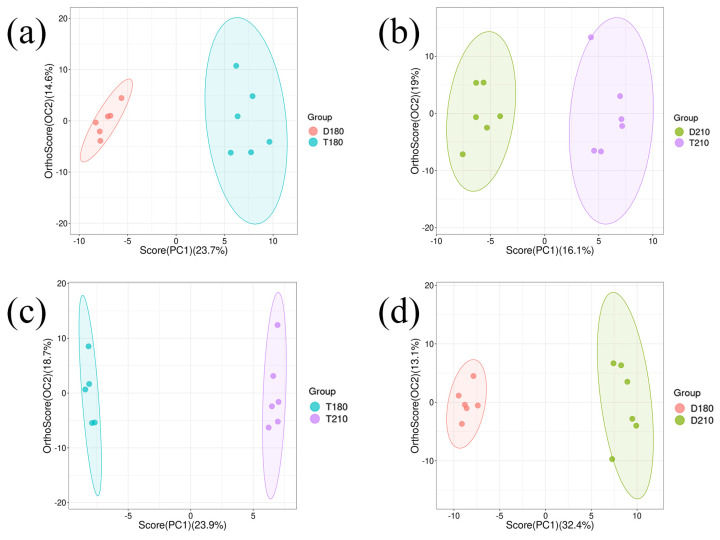
OPLS-DA analysis of volatile flavor substance content in LD muscle of each group (**a**) D180 and T180; (**b**) D210 and T210; (**c**) T180 and T210; (**d**) D180 and D210. D180, T180, D210 and T210 represent 180-day-old Duroc, 180-day-old TB pig, 210-day-old Duroc and 210-day-old TB pig, respectively.

Systematic data preprocessing and statistical analysis were performed for 1518 volatile flavor compounds. Firstly, all compounds were normalized by the internal normalization method, and quantitative correction was performed according to the peak area ratio of each compound to the internal standard to eliminate systematic errors caused by concentration differences between samples. Subsequently, a rigorous quality control step was used to eliminate compounds that were not detected in more than 50% of the samples, resulting in 249 statistically significant volatile flavor substances (Supplementary Data S2). OPLS-DA offers significant advantages in sample classification, feature extraction, and data dimensionality reduction. Figure 4 effectively demonstrates the classification of pork samples, with distinct differences observed in each group.

#### 3.5.5. Differential Compounds Analysis

**Figure 5:**
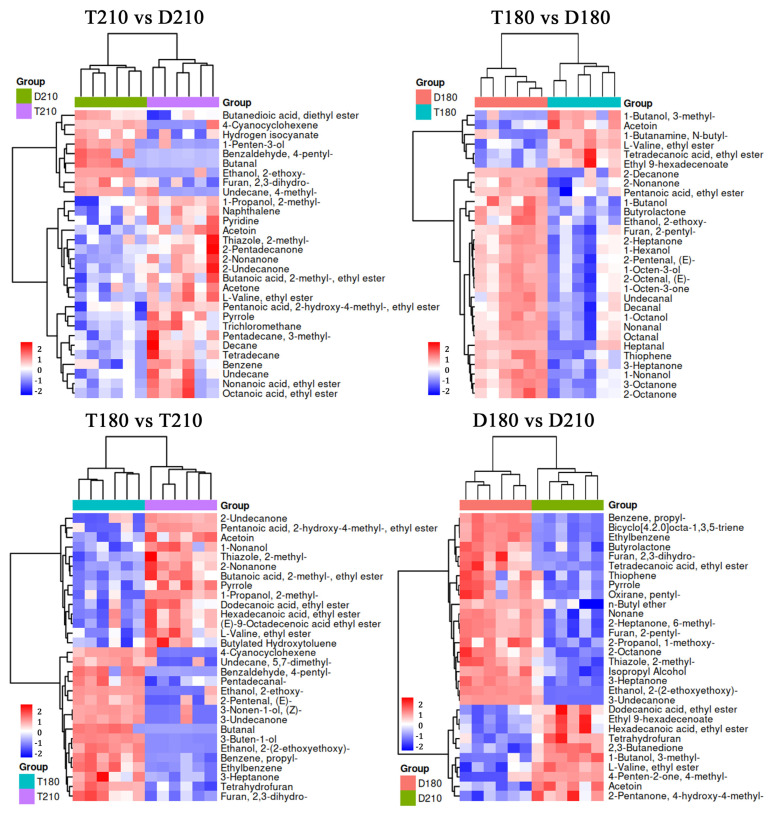
Differences in volatile compounds between groups. D180, T180, D210 and T210 represent 180-day-old Duroc, 180-day-old TB pig, 210-day-old Duroc and 210-day-old TB pig, respectively.

*T* test (*p* < 0.05) and VIP > 1 were used to screen the differential flavor compounds with flavor characteristics. The VIP value reflects the degree to which each variable contributes to sample classification in the OPLS-DA model, according to the VIP value, the top 30 differential substances were selected. As shown in Figure 5, compared with Duroc pig, L-Valine ethyl ester and Acetoin were up-regulated, while 2-ethoxy-Ethanol was down-regulated in Taoyuan black pig. Compared with 210-day-old pigs, propyl-Benzene and 3-Heptanone were up-regulated, Acetoin and L-Valine ethyl ester were down-regulated in 180-day-old pigs.

## 4. Discussion

In this study, we comprehensively compare the differences in meat quality and flavor quality between the two different breeds. Meat quality, as a complex trait, can be evaluated through various indicators, especially sensory quality including pH, meat color, dripping loss, IMF, and shear force [22]. The L*, a*, and b* values are standard measures of pork color, with lower L* values, higher a* values, and lower b* values indicating richer myoglobin content in the muscle and fresher meat [23]. This indicates that there are significant differences in meat color between different ages and breeds. In the present study, as pigs’ age, TB pork exhibits better muscle color and higher tenderness compared to Duroc, making them potentially more appealing to consumers. Meat with a pH_45min_ value being above 6.0 and a pH_24h_ value ranging between 5.5 and 5.7 indicate that the meat quality is within the ideal range [24]. The water-holding capacity, cooking loss, and dripping loss of pork are economically significant factors in the food processing industry. In this study, the dripping loss and cooking loss of LD muscle increased with age in both breeds of Duroc and TB pigs. The results of this study suggest that among the four groups, 180-day-old TB pigs were more cost-effective for enterprises. Pork with lower moisture content often exhibits better meat quality and stronger chewiness, which is negatively correlated with IMF [25]. The accumulation of IMF creates a “marbling” effect in the muscle, impacting sensory quality attributes of pork such as tenderness, flavor, and juiciness [26]. As an introduced breed, the IMF value of Duroc pig is usually lower than that of local pig breeds in China. Li et al. [27] suggested that the IMF value of Duroc pig LD muscle was lower than that of Chinese Huai pigs. Therefore, our results confirm that the superior meat quality of TB pigs is characterized by low moisture content and high IMF.

The response values of the electronic tongue are crucial for evaluating meat taste quality, which primarily encompasses five basic taste attributes: umami, saltiness, sourness, bitterness, and astringency. These taste characteristics are mainly mediated by water-soluble flavor compounds [28]. Compared with the same-aged Duroc pigs, TB pigs showed a higher umami content, which may be related to their higher intramuscular fat content. Many reports have pointed out that the higher the IMF value of pork, the more delicious the meat is [29]. As previous research pointed out, the older the DLY finishing pigs, the lower their sensory quality [3]. The period around 180 days of age could be a crucial ‘window stage’ for the formation of pork quality.

Fatty acids are important fat-soluble flavor precursors in pork, and their composition affects pork flavor. The results of this experiment manifested that C16:0, C18:0, C18:1n9c, and C18:2n6 are the primary fatty acids in the meat of the two pig breeds, with their levels higher than these of other fatty acids, and it was consistent with previous research [30]. Fatty acid composition is significantly influenced by breed factors, with saturated and monounsaturated fatty acids both higher in TB pigs compared to that of Duroc pigs, especially oleic acid (C18:1n9c). In contrast, polyunsaturated fatty acids C18:2n6, C18:3n3, C20:3n6, and C20:4n6 are significantly lower in TB pigs compared to that of Duroc pigs. Chinese black pigs usually had higher rates of monounsaturated fatty acids and lower levels of polyunsaturated fatty acid [31]. Some studies have reported that C18:2n6, C18:3n3, C20:3n6, and C20:4n6 are negatively correlated with pork flavor and consumer acceptability, while C18:1n9c shows a positive impact, and plays a significant role in flavor [32]. This may be one of the reasons why the meat flavor of TB pig is popular. C18:1n9c is a type of monounsaturated fatty acids. Besides contributing to pork flavor, it also has been linked to increased lifespan when consumed in higher quantities in human diets [33]. This suggests that selecting TB pork for human consumption may have a positive impact on health. Furthermore, according to nutritional recommendations, a higher ratio of n-6 to n-3 polyunsaturated fatty acids in human diets increases the risk of cancer [33]. In this experiment, the n-6: n-3 ratio of TB was significantly lower than that of Duroc. In addition, the n-6: n-3 ratio of pork decreases over time, and TB pork or older pork has higher nutritional value, which is beneficial for human dietary health in the long term.

The flavor during the hydrolysis of muscle proteins primarily comprises two aspects: taste and aroma [34], and taste is significantly influenced by nucleotides and free amino acids. Nucleotides such as GMP, XMP, and IMP are well-known flavor enhancers. The present experimental results showed that the contents of GMP and IMP in TB pigs were higher than Duroc pigs. Amino acids can affect the taste and flavor of meat by providing nutritional value and flavor characteristics [35], and amino acids can be categorized based on their flavor into umami amino acids, sweet amino acids, and bitter amino acids [36]. Our research found that, except for Ser and Asp, other amino acids are influenced by the interaction of age and breed factors, indicating that flavor amino acids are not determined by a single factor. Regarding the breed, the LD muscle of TB pigs has higher contents of umami amino acids, sweet amino acids, and total amino acids compared to that of Duroc pigs, suggesting a richer taste profile in the former breed. Concerning slaughter age, the meat of the pigs at 180 days has higher levels of umami amino acids, sweet amino acids, and total amino acids compared to that of 210 days, indicating better taste at the age of 180 days.

Limited study on the flavor compounds discrepancy in the muscle between Duroc and TB pigs, and comprehensive two-dimensional GC×GC TOF MS analysis was used in this experiment. It offers higher throughput, precision, and sensitivity compared to traditional GC-MS, enabling the identification of a greater number of flavor compounds. This study showed that there were more flavor substances in local Chinese black pigs, and the flavor substances gradually decreased with increase in age. Aldehydes and aromatic compounds contained in pork had a significant effect on flavor [36]. The predominance of hetercyclic compounds and aromatic compounds, in our results clearly demonstrates the thermal origin of these markers. The increase in temperature will continuously raise the content and diversity of volatile compounds, forming a unique aroma [37]. These suggests that TB pigs, which contained more aldehydes and aromatic compounds, were more popular among consumers. We performed a quantitative analysis using the internal standard normalization method and detected significant levels of alcohols, such as 1-hexanol (green), 1-octen-3-ol (mushroom), ethanol (alcoholic), 1-pentanol (vanilla), and 2-ethylhexanol (rose, green). Aldehydes were found to have a more substantial impact on flavor. Among the aldehydes, hexanal (green grass, fruity) had the highest concentration in the overall pork samples, primarily arising from the oxidative breakdown of the main fatty acids in pork. Previous studies have confirmed that hexanal is significantly associated with fatty acids such as C16:0 palmitic acid, C18:1n9c oleic acid, and C18:2n6c linoleic acid [9].

However, due to the varying concentrations and different odor thresholds of these volatile compounds, it is not possible to directly determine the primary contributors to flavor. ROAV is an effective standard for flavor assessment, which can effectively identify the primary contributors to the detectable flavor. A compound is identified as a key flavor-active substance if its ROAV is 1 or higher. We established the ROAV of the compound with the highest aroma threshold as 100. The ROAVs for other flavor compounds were determined using a formula based on their respective concentrations. In the present study, the most prominent flavor compounds in pork were 2-nonenal (fatty, cucumber), 2-octenal (nuts, green, fatty), heptanal (citrus, fatty, rancid), 2,3-butanedione (pleasant, buttery), and 2-pentylfuran (pleasant, buttery). These compounds were present across all four groups, indicating that they are common flavor contributors to pork. PUFAs, which produce furans during heating, may be due to the high concentration of 2-pentylfuran in Duroc pigs due to their high polyunsaturated fatty acids.

TB pork contained higher levels of 2-ethylhexanol and 2,3-butanedione. Yuan et al. [16] demonstrated that 2,3-butanedione is one of the core volatile flavor compounds in marinated pork, exhibiting the highest ROAV. This diketone plays a crucial role in the formation of sweet, fruity, and caramel-like flavor characteristics. This result may correlate with the free amino acid content, as the higher free amino acid levels in the muscle of TB pigs contribute to the abundance of unique flavor compounds. Although Duroc pork had higher levels of 2-nonenal, 2-octenal, and 1-octen-3-one (mushroom-like), it also had high concentrations of heptanal and pentanal (sickening, rancid, decayed). Previous study reported that when the concentration of pentanal is high, it is typically described as having an unpleasant taste [38,39]. These findings suggest that heptanal and pentanal may be obstacles to consumer acceptance of the meat of Duroc pigs and affect the overall flavor of the pork. 1-Octen-3-one, which primarily imparts a mushroom flavor, is easily reduced to 1-octen-3-ol and can produce an unpleasant odor at high concentrations [9,28]. Studies have shown that during pork refrigeration, the meat is more prone to spoilage due to high protease activity, leading to the accumulation of volatile compounds such as 1-octen-3-one [40]. These findings are in line with our experimental observations. Furthermore, although some volatile compounds have high thresholds, they can produce synergistic effects, making it crucial to compare the different flavor compounds between the two pig breeds. OPLS-DA was able to distinguish volatile flavor compounds from each group, we observed that the ester compounds in the TB pigs were significantly higher than Duroc pigs, imparting a unique aromatic or fruity scent. Esters are created when free fatty acids in muscle tissue combine with alcohols [41]. High content of free amino acids in the meat of TB pigs might be one of the reasons to produce many ester compounds. L-Valine ethyl ester in pork may be associated with MUFAs, especially oleic acid because of their consistent trend. It decreases with age. Action (creamy, milky) is a key volatile flavor compound and an important flavor enhancer, which is positively related to the content of IMP and 2,3-butanedione in the pork [42,43]. These volatile compounds contribute positively to flavor profiles and represent significantly differentiated volatile components between TB pigs and Duroc pigs. These findings suggest that pork flavor is influenced by multiple factors and that flavor variation in pork during different growth stages provides potential opportunities for producers to regulate flavor. 2-Ethoxy-ethanol (sweet, musty) belongs to the ether class, while it might seem like it could have a pleasant fragrance in some contexts, it is generally considered an unpleasant odor in food products like meat. This is because, in high concentrations, it can impart an off-putting or undesirable flavor profile. High levels of 2-Ethoxy-ethanol in Duroc pigs may lead to barriers to consumer acceptance.

In addition, Propyl-Benzene and 3-Heptanone (fruity, fatty) were found to increase with aging in both breeds, indicating that flavor formation is complex and needs to be analyzed in combination with specific compounds. The content of these key flavor compounds can be optimized by pig age, or selection of specific breeds to improve the flavor of pork and the consumer experience. At the same time, the in-depth study of pork flavor compounds can also provide new ideas for the food industry in the flavoring and processing process.

## 5. Conclusions

In summary, TB pigs exhibited lower dripping loss, cooking loss, and shear force and higher IMF, flavor nucleotides, free amino acids, and monounsaturated fatty acid content than Duroc pigs. Electronic tongue analysis revealed that the highest umami values were observed in 180-day-old TB pigs. GC×GC-TOF-MS analysis showed that TB pigs contained more flavoring substances, aldehydes, and aromatic compounds. ROAV analysis identified the key volatile compounds in the meat of the two pig breeds to be 2-nonenal, 2-octenal, heptanal, 2,3-butanedione, and 2-pentylfuran. We also performed a comparative analysis of the different volatile flavor compounds. This study enhances the research on the flavor of indigenous Chinese pig breeds and provides a theoretical basis for breeding and nutritional regulation.

## Figures and Tables

**Table 1:** Comparison of meat quality and chemical composition of LD muscles in pigs of different ages and breeds.

Item	180-Day-Old		210-Day-Old		SEM	*p*-Values		
	T180	D180	T210	D210		*p*_A_	*p*_B_	*p*_A*B_
pH_45min_	6.61	6.44	6.41	6.07	0.04	<0.01	<0.01	0.33
pH_24h_	5.55	5.56	5.69	5.76	0.02	<0.01	0.96	0.93
L*	51.92	52.60	51.59	49.44	0.28	<0.01	0.20	0.02
a*	15.69	14.79	16.46	16.38	0.15	<0.01	0.12	0.19
b*	6.11	6.90	8.02	7.80	0.21	<0.01	0.51	0.24
Shear force, N	67.66	81.31	61.76	75.30	2.70	0.28	0.02	0.99
Cooking loss, %	20.71	23.64	25.17	28.13	0.42	<0.01	<0.01	0.99
Dripping loss, %	3.19	3.95	3.86	4.42	0.10	0.01	<0.01	0.62
Water holding capacity, %	62.79	61.16	68.24	61.67	1.01	0.15	0.05	0.23
Moisture, %	71.80	73.10	72.47	74.70	0.19	0.01	<0.01	0.23
Crude protein, %	23.38	23.44	23.06	22.98	0.13	0.15	0.98	0.79
Intramuscular fat, %	3.91	2.38	3.34	2.01	0.19	0.23	<0.01	0.80

Note: D180, T180, D210 and T210 represent 180-day-old Duroc, 180-day-old TB pig, 210-day-old Duroc and 210-day-old TB pig, respectively. A, age; B, breed; A*B, age*breed. *n* = 10.

**Table 2:** Comparison of response values of electronic tongue sensors in LD muscles of pigs of different ages and breeds.

Item	180-Day-Old		210-Day-Old		SEM	*p*-Values		
	T180	D180	T210	D210		*p*_A_	*p*_B_	*p*_A*B_
Sourness	−32.26	−32.06	−35.70	−37.14	0.33	<0.01	0.36	0.23
Bitterness	0.34	1.52	−0.67	−0.56	0.07	<0.01	<0.01	<0.01
Astringency	−1.58	−1.47	−2.14	−2.26	0.06	<0.01	0.97	0.34
Umami	12.56	12.17	12.25	12.09	0.08	0.22	0.08	0.46
Richness	3.97	3.80	2.93	2.81	0.05	<0.01	0.16	0.84
Saltiness	−10.53	−10.24	−9.46	−8.93	0.14	<0.01	0.15	0.67

Note: D180, T180, D210 and T210 represent 180-day-old Duroc, 180-day-old TB pig, 210-day-old Duroc and 210-day-old TB pig, respectively. A, age; B, breed; A*B, age*breed. *n* = 10.

**Table 3:** Comparison of free amino acids in LD muscle of pigs of different ages and breeds.

Item, µg/g	180-Day-Old		210-Day-Old		SEM	*p*-Values		
	T180	D180	T210	D210		*p*_A_	*p*_B_	*p*_A*B_
Umami amino acids								
Asp	9.90	7.23	10.21	10.32	0.52	0.11	0.23	0.19
Glu	124.80	86.57	29.57	29.27	3.02	<0.01	<0.01	<0.01
Sweet amino acids								
Ser	82.72	64.62	46.73	40.79	2.03	<0.01	0.01	0.14
Gly	128.24	139.54	144.11	125.25	3.24	0.90	0.56	0.03
Thr	78.83	59.50	29.93	28.03	1.14	<0.01	<0.01	<0.01
Ala	267.04	225.33	178.26	158.77	3.51	<0.01	<0.01	0.12
Bitter amino acids								
His	28.06	18.40	15.30	15.02	0.53	<0.01	<0.01	<0.01
Tyr	67.13	51.29	30.88	34.69	1.21	<0.01	0.02	<0.01
Ile	49.84	35.10	19.53	19.74	0.73	<0.01	<0.01	<0.01
Leu	100.92	65.39	42.12	45.73	1.40	<0.01	<0.01	<0.01
Phe	61.09	43.82	25.81	26.14	0.90	<0.01	<0.01	<0.01
Trp	13.72	9.23	4.56	5.44	0.25	<0.01	<0.01	<0.01
Other amino acids								
Arg	109.64	79.45	42.13	38.73	1.79	<0.01	<0.01	<0.01
Pro	38.38	28.78	22.12	23.02	1.11	<0.01	0.06	0.02
Cys	4.60	4.37	6.33	3.74	0.26	0.30	0.01	0.03
Lys	68.43	44.37	28.74	27.91	1.13	<0.01	<0.01	<0.01
Met	43.95	32.00	11.89	12.75	0.59	<0.01	<0.01	<0.01
Val	68.93	48.20	30.46	31.13	1.13	<0.01	<0.01	<0.01
Total	1380.29	996.58	673.91	634.28	16.61	<0.01	<0.01	<0.01

Note: D180, T180, D210 and T210 represent 180-day-old Duroc, 180-day-old TB pig, 210-day-old Duroc and 210-day-old TB pig, respectively. A, age; B, breed; A*B, age*breed. *n* = 10.

**Table 4:** Comparison of flavor nucleotide differences in LD muscle of pigs of different ages and breeds.

Item, μg/g	180-Day-Old		210-Day-Old		SEM	*p*-Values		
	T180	D180	T210	D210		*p*_A_	*p*_B_	*p*_A*B_
GMP	40.92	32.89	41.96	32.89	1.19	0.83	<0.01	0.83
IMP	1740.38	1713.20	1890.50	1671.73	44.63	0.55	0.18	0.29
AMP	135.16	83.45	102.62	96.96	4.36	0.29	<0.01	0.01

Note: D180, T180, D210 and T210 represent 180-day-old Duroc, 180-day-old TB pig, 210-day-old Duroc and 210-day-old TB pig, respectively. A, age; B, breed; A*B, age*breed. *n* = 10. GMP, Guanosine Monophosphate; IMP, Inosine Monophosphate. AMP, Adenosine Monophosphate.

**Table 5:** Comparison of fatty acids in LD muscle of pigs of different ages and breeds.

Item	180-Day-Old		210-Day-Old		SEM	*p*-Values		
	T180	D180	T210	D210		*p*_A_	*p*_B_	*p*_A*B_
Saturated fatty acid								
C10:0	0.13	0.13	0.12	0.06	0.01	<0.01	0.02	0.02
C12:0	0.13	0.11	0.12	0.06	0.01	0.03	0.02	0.10
C14:0	1.75	1.51	1.65	1.54	0.03	0.49	<0.01	0.19
C16:0	27.94	26.27	27.68	25.58	0.17	0.17	<0.01	0.54
C17:0	0.18	0.23	0.16	0.21	0.00	0.04	<0.01	0.75
C18:0	15.25	15.10	14.28	14.52	0.22	0.08	0.91	0.66
C20:0	0.23	0.20	0.25	0.20	0.00	0.13	<0.01	0.23
∑SFA	45.56	43.41	44.13	42.03	0.32	0.03	<0.01	0.97
Monounsaturated fatty acid								
C16:1	3.31	3.16	3.49	3.12	0.08	0.65	0.11	0.51
C18:1n9t	0.13	0.15	0.13	0.12	0.00	<0.01	0.21	<0.01
C18:1n9c	37.28	35.15	39.24	36.98	0.37	0.02	0.01	0.93
C20:1	0.67	0.57	0.75	0.62	0.02	0.04	<0.01	0.72
∑MUFA	41.39	39.03	43.59	40.84	0.44	0.03	0.01	0.83
Polyunsaturated fatty acid								
C18:2n6c	10.46	13.94	9.99	13.76	0.33	0.62	<0.01	0.82
C18:3n3	0.44	0.49	0.46	0.56	0.01	0.05	<0.01	0.20
C20:2	0.36	0.44	0.37	0.50	0.01	0.06	<0.01	0.19
C20:3n6	0.20	0.28	0.18	0.27	0.01	0.51	<0.01	0.72
C20:4n6	1.57	2.40	1.25	2.03	0.09	0.06	<0.01	0.92
∑PUFA	13.03	17.56	12.25	17.13	0.42	0.85	<0.01	0.78
n-3PUFAs	0.44	0.49	0.46	0.56	0.01	0.05	<0.01	0.20
n-6PUFAs	12.24	16.62	11.42	16.07	0.41	0.41	<0.01	0.87
n-6: n-3	27.85	33.72	24.80	28.82	0.77	0.02	<0.01	0.56

Note: D180, T180, D210 and T210 represent 180-day-old Duroc, 180-day-old TB pig, 210-day-old Duroc and 210-day-old TB pig, respectively. A, age; B, breed; A*B, age*breed. *n* = 10. n-3 PUFAs = C18:3n3; n-6 PUFA = C18:2n6c + C20:3n6 + C20:4n6.

**Table 6:** Comparison of ROAVs of volatile flavor compounds in LD muscle of pigs of different ages and breeds.

Item	Class	T180 ROAVs	D180 ROAVs	T210 ROAVs	D210 ROAVs	Odor Character
1-Hexanol, 2-ethyl-	Alcohols	1.34	1.00	<1.00	<1.00	Rose, Green
2-Nonenal, (E)-	Aldehydes	47.87	81.55	3.81	5.62	Fatty, Cucumber
2-Octenal, (E)-	Aldehydes	12.57	33.63	1.37	3.99	Nuts, Green, Fatty
Pentanal	Aldehydes	1.22	2.10	<1.00	<1.00	sickening, rancid, decayed
Heptanal	Aldehydes	15.01	86.67	2.78	12.51	Citrus, Fatty, Rancid
1-Octen-3-one	Ketones	7.22	14.57	<1.00	<1.00	Mushroom-Like
2,3-Butanedione	Ketones	100.00	60.51	100.00	100.00	pleasant, buttery
Furan, 2-pentyl-	Organo heterocyclic compounds	40.76	97.94	3.51	3.36	Green Beans, Vegetable

Note: In the table, “<1.00” means that the ROAV of the compound is <1.

## Data Availability

The original contributions presented in the study are included in the article/Supplementary Material, further inquiries can be directed to the corresponding authors.

## Supplementary Materials

The following supporting information can be downloaded at: https://www.mdpi.com/article/10.3390/foods14111935/s1.

## Abbreviations

The following abbreviations are used in this manuscript:

AMP	Adenosine Monophosphate
GC×GC-TOF MS	Gas Chromatography-Time-of-Flight Mass Spectrometry
GMP	Guanosine Monophosphate
IMF	Intramuscular Fat
IMP	Inosine Monophosphate
LD	Longissimus Dorsi
MUFA	Monounsaturated Fatty Acid
OPLS-DA	Orthogonal Partial Least Squares-Discriminant Analysis
pH	Potential of Hydrogen
PUFA	Polyunsaturated Fatty Acid
ROAV	Relative Odor Activity Value
VIP	Variable Importance in Projection

## References

[1] Miller R. (2020). Drivers of Consumer Liking for Beef, Pork, and Lamb: A Review. Foods.

[2] Bonneau M., Lebret B. (2010). Production systems and influence on eating quality of pork. Meat Sci..

[3] Guo Z., Chen X., Chen D., Li M., Yin J., Yu B., He J., Huang Z. (2022). Effects of slaughter age on carcass traits and meat quality of crossbred (Duroc × Landrace × Yorkshire) finishing pigs. Anim. Biotechnol..

[4] Duan Y., Zheng C., Zheng J., Ma L., Ma X., Zhong Y., Zhao X., Li F., Guo Q., Yin Y. (2023). Profiles of muscular amino acids, fatty acids, and metabolites in Shaziling pigs of different ages and relation to meat quality. Sci. China Life Sci..

[5] Kucha C.T., Liu L., Ngadi M.O. (2018). Non-Destructive Spectroscopic Techniques and Multivariate Analysis for Assessment of Fat Quality in Pork and Pork Products: A Review. Sensors.

[6] Choi Y.S., Hwang K.E., Kim H.W., Song D.H., Jeon K.H., Park J.D., Sung J.M., Kim Y.B., Kim C.J. (2016). Replacement of Pork Meat with Pork Head Meat for Frankfurters. Korean J. Food Sci. Anim. Resour..

[7] Zhang J., Meng S., Wang H., Zhang C., Sun Z., Huang L., Miao Z. (2024). Comparison of Growth Performance, Carcass Properties, Fatty Acid Profile, and Genes Involved in Fat Metabolism in Nanyang and Landrace Pigs. Genes.

[8] Razmaite V., Sveistiene R., Siukscius A. (2024). Effects of Genotype on Pig Carcass, Meat Quality and Consumer Sensory Evaluation of Loins and Bellies. Foods.

[9] Wu W., Zhan J., Tang X., Li T., Duan S. (2022). Characterization and identification of pork flavor compounds and their precursors in Chinese indigenous pig breeds by volatile profiling and multivariate analysis. Food Chem..

[10] Zhang Y., Zhang Y.J., Li H., Guo T.R., Jia J.L., Zhang P.C., Wang L.G., Xia N., Qian Q., Peng H.C. (2022). Comparison of Nutrition and Flavor Characteristics of Five Breeds of Pork in China. Foods.

[11] Ngapo T.M., Gariepy C. (2008). Factors affecting the eating quality of pork. Crit. Rev. Food Sci. Nutr..

[12] Song B., Cheng Y., Azad M.A.K., Ding S., Yao K., Kong X. (2023). Muscle characteristics comparison and targeted metabolome analysis reveal differences in carcass traits and meat quality of three pig breeds. Food Funct..

[13] Ding S., Cheng Y., Azad M.A.K., Zhu Q., Huang P., Kong X. (2022). Developmental Changes of Immunity and Different Responses to Weaning Stress of Chinese Indigenous Piglets and Duroc Piglets during Suckling and Weaning Periods. Int. J. Mol. Sci..

[14] National Research Council (NRC) (2012). Nutrient Requirements of Swine.

[15] Zhao X., Feng J., Laghi L., Deng J., Dao X., Tang J., Ji L., Zhu C., Picone G. (2023). Characterization of Flavor Profile of “Nanx Wudl” Sour Meat Fermented from Goose and Pork Using Gas Chromatography-Ion Mobility Spectrometry (GC-IMS) Combined with Electronic Nose and Tongue. Foods.

[16] Yuan H.B., Wu H.C., Qiao M.F., Tang W.T., Dong P., Deng J. (2024). Characterization of Flavor Profile of Sauced Pork from Different Regions of China Based on E-Nose, E-Tongue and Gas Chromatography-Ion Mobility Spectroscopy. Molecules.

[17] Cody R.B., Sparkman O.D., Moore H. (2022). Creating a Searchable Chromatographic Database with the NIST Mass Spectral Search Program. J. Am. Soc. Mass. Spectrom..

[18] Djoumbou Feunang Y., Eisner R., Knox C., Chepelev L., Hastings J., Owen G., Fahy E., Steinbeck C., Subramanian S., Bolton E. (2016). ClassyFire: Automated chemical classification with a comprehensive, computable taxonomy. J. Cheminform..

[19] Dashti H., Wedell J.R., Westler W.M., Markley J.L., Eghbalnia H.R. (2019). Analysis: Automated evaluation of consistency within the PubChem Compound database. Sci. Data.

[20] Vass A.A., Smith R.R., Thompson C.V., Burnett M.N., Wolf D.A., Synstelien J.A., Dulgerian N., Eckenrode B.A. (2004). Decompositional odor analysis database. J. Forensic Sci..

[21] Garg N., Sethupathy A., Tuwani R., Nk R., Dokania S., Iyer A., Gupta A., Agrawal S., Singh N., Shukla S. (2018). FlavorDB: A database of flavor molecules. Nucleic Acids Res..

[22] Schwab C.R., Baas T.J., Stalder K.J., Mabry J.W. (2006). Effect of long-term selection for increased leanness on meat and eating quality traits in Duroc swine. J. Anim. Sci..

[23] Liu Q., Long Y., Zhang Y.F., Zhang Z.Y., Yang B., Chen C.Y., Huang L.S., Su Y. (2021). Phenotypic and genetic correlations of pork myoglobin content with meat colour and other traits in an eight breed-crossed heterogeneous population. Animal.

[24] Matarneh S.K., Silva S.L., Gerrard D.E. (2021). New Insights in Muscle Biology that Alter Meat Quality. Annu. Rev. Anim. Biosci..

[25] Gao G., Gao N., Li S., Kuang W., Zhu L., Jiang W., Yu W., Guo J., Li Z., Yang C. (2021). Genome-Wide Association Study of Meat Quality Traits in a Three-Way Crossbred Commercial Pig Population. Front. Genet..

[26] Yu T.Y., Tian X.K., Li D., He Y.L., Yang P.Y., Cheng Y., Zhao X., Sun J.C., Yang G.S. (2023). Transcriptome, proteome and metabolome analysis provide insights on fat deposition and meat quality in pig. Food Res. Int..

[27] Li X.J., Lu L.Y., Tong X.W., Li R.D., Jin E., Ren M., Gao Y.F., Gu Y.F., Li S.H. (2023). Transcriptomic Profiling of Meat Quality Traits of Skeletal Muscles of the Chinese Indigenous Huai Pig and Duroc Pig. Genes.

[28] Wang Y., Liu X., Wang Y., Zhao G., Wen J., Cui H. (2022). Metabolomics-Based Analysis of the Major Taste Contributors of Meat by Comparing Differences in Muscle Tissue between Chickens and Common Livestock Species. Foods.

[29] Zheng C., Wan M., Guo Q., Duan Y., Yin Y. (2025). Glutamate increases the lean percentage and intramuscular fat content and alters gut microbiota in Shaziling pigs. Anim. Nutr..

[30] Duan S., Tian Z., Zheng X., Tang X., Li W., Huang X. (2024). Characterization of flavour components and identification of lipid flavour precursors in different cuts of pork by phospholipidomics. Food Chem..

[31] Chen Q., Zhang W., Xiao L., Sun Q., Wu F., Liu G., Wang Y., Pan Y., Wang Q., Zhang J. (2023). Multi-Omics Reveals the Effect of Crossbreeding on Some Precursors of Flavor and Nutritional Quality of Pork. Foods.

[32] Cameron N.D., Enser M., Nute G.R., Whittington F.M., Penman J.C., Fisken A.C., Perry A.M., Wood J.D. (2000). Genotype with nutrition interaction on fatty acid composition of intramuscular fat and the relationship with flavour of pig meat. Meat Sci..

[33] Papsdorf K., Miklas J.W., Hosseini A., Cabruja M., Morrow C.S., Savini M., Yu Y., Silva-Garcia C.G., Haseley N.R., Murphy L.M. (2023). Lipid droplets and peroxisomes are co-regulated to drive lifespan extension in response to mono-unsaturated fatty acids. Nat. Cell Biol..

[34] Maughan C., Tansawat R., Cornforth D., Ward R., Martini S. (2012). Development of a beef flavor lexicon and its application to compare the flavor profile and consumer acceptance of rib steaks from grass- or grain-fed cattle. Meat Sci..

[35] Ma X., Yu M., Liu Z., Deng D., Cui Y., Tian Z., Wang G. (2020). Effect of amino acids and their derivatives on meat quality of finishing pigs. J. Food Sci. Technol..

[36] Chen D.-W., Zhang M. (2007). Non-volatile taste active compounds in the meat of Chinese mitten crab (*Eriocheir sinensis*). Food Chem..

[37] Wang X., Wang X., Zhang X., Liu S., Yu J., Cui H., Xia S., Ho C.T. (2023). Changes of lipid oxidation, volatile and taste-active compounds during pan-heating of pork belly. Food Res. Int..

[38] Coombs C.E.O., Holman B.W.B., Ponnampalam E.N., Morris S., Friend M.A., Hopkins D.L. (2018). Effects of chilled and frozen storage conditions on the lamb *M. longissimus lumborum* fatty acid and lipid oxidation parameters. Meat Sci..

[39] Liu Z., Huang Y., Kong S., Miao J., Lai K. (2023). Selection and quantification of volatile indicators for quality deterioration of reheated pork based on simultaneously extracting volatiles and reheating precooked pork. Food Chem..

[40] Sun G., Yang J., Holman B.W.B., Tassou C.C., Papadopoulou O.S., Luo X., Zhu L., Mao Y., Zhang Y. (2024). Exploration of the shelf-life difference between chilled beef and pork with similar initial levels of bacterial contamination. Meat Sci..

[41] Hou M., Liu D., Xu X., Zhou G., Li C. (2018). Effect of postmortem aging time on flavor profile of stewed pork rib broth. Int. J. Food Prop..

[42] Pongsetkul J., Yongsawatdigul J., Boonanuntanasarn S., Benjakul S. (2022). Development of Flavor and Taste Components of Sous-Vide-Cooked Nile Tilapia (*Oreochromis niloticus*) Fillet as Affected by Various Conditions. Foods.

[43] Yan X., Pan S., Liu X., Tan M., Zheng X., Du W., Wu M., Song Y. (2023). Profiling the Major Aroma-Active Compounds of Microwave-Dried Jujube Slices through Molecular Sensory Science Approaches. Foods.

